# The whole treatment process and thinking of a patient with NUT carcinoma of the parotid gland: a case report

**DOI:** 10.3389/fonc.2023.1094770

**Published:** 2023-05-02

**Authors:** Shujuan Fu, Zhiying Wang, Cunya Li, Yun Li, Ke Zhang, Zhixian Zhong, Yi Zhong

**Affiliations:** ^1^ Oncology Department, Shanghai TCM-Intergrated Hospital Shanghai University of Traditional Chinese Medicine, Shanghai, China; ^2^ Graduate School, Shanghai University of Traditional Chinese Medicine, Shanghai, China; ^3^ Breast Cancer Center, East Hospital Affiliated to Tongji University, Tongji University School of Medicine, Tongji University, Shanghai, China

**Keywords:** NUT carcinoma, immunotherapy, targeted therapy, case report, overall survival (OS)

## Abstract

**Background:**

Primary nuclear protein in testis (NUT) carcinoma is a rare malignant tumor originating from the salivary glands that usually occurs in midline structures, such as the head and neck, and has been identified in young patients. Progression of NUT carcinoma is rapid, and there is a high degree of malignant invasion. The median survival time of NUT carcinoma patients is 6 to 9 months, and 80% of the patients die within 1 year after diagnosis.

**Case description:**

This case report summarizes the treatment of a 36-year-old male patient with NUT carcinoma of the right parotid gland. The overall survival of the patient was 2 years. We also discuss the applications and outcomes of immune checkpoint inhibitor and targeted therapy combination regimens in the treatment of NUT carcinoma.

**Conclusion:**

We suggest that targeted therapy combined with immunotherapy which has long-term clinical benefits and targeted therapy which has high clinical response rate(immunotherapy + dual-targeting three-drug regimens) is an ideal choice for the treatment of patients with rare and/or refractory tumors and will not compromise patient safety.

**Clinical trial registration:**

identifier ChiCTR1900026300.

## 1 Introduction

Nuclear protein in testis (NUT) carcinoma was first discovered and reported by Kees et al. (1) in 1991, and the case included a mediastinal/thymic tumor. NUT carcinoma is a malignant tumor caused by rearrangement of the NUT gene on chromosome 15: NUTM1. In the majority of cases (~75%), NUTM1 is fused to BRD4. Many other fusion partners exist, including BRD3, NSD3, ZNF532, and ZNF592, and the resulting fusion genes encode BRD4-interacting proteins that serve to link NUT with BRD4, essentially forming the same oncogenic complex as that of BRD4-NUT (2–5). NUT carcinoma is more common in the midline of the larynx, nasal cavity, and mediastinum. It can also be seen in the liver, pancreas, bladder and other organs (6). Primary NUT carcinoma of the salivary gland is very rare, with only 15 such cases reported in the literature to date (7). NUT carcinoma progresses rapidly and has a high degree of malignant invasion, with a median survival time of 6-9 months.

No established treatment protocol exists for the disease, although some success has been achieved using a multimodal approach, including early surgical resection and adjuvant chemotherapy and radiation. Here, we report the whole treatment process of a patient with right parotid NUT carcinoma and summarize the clinicopathologic diagnosis (8). The overall survival (OS) of this patient was 2 years. The applications and outcomes of immune checkpoint inhibitors combined with targeted therapy in the treatment of NUT carcinoma are also discussed.

## 2 Case presentation

### 2.1 Medical records

#### 2.1.1 Basic information

**Figure 1:**
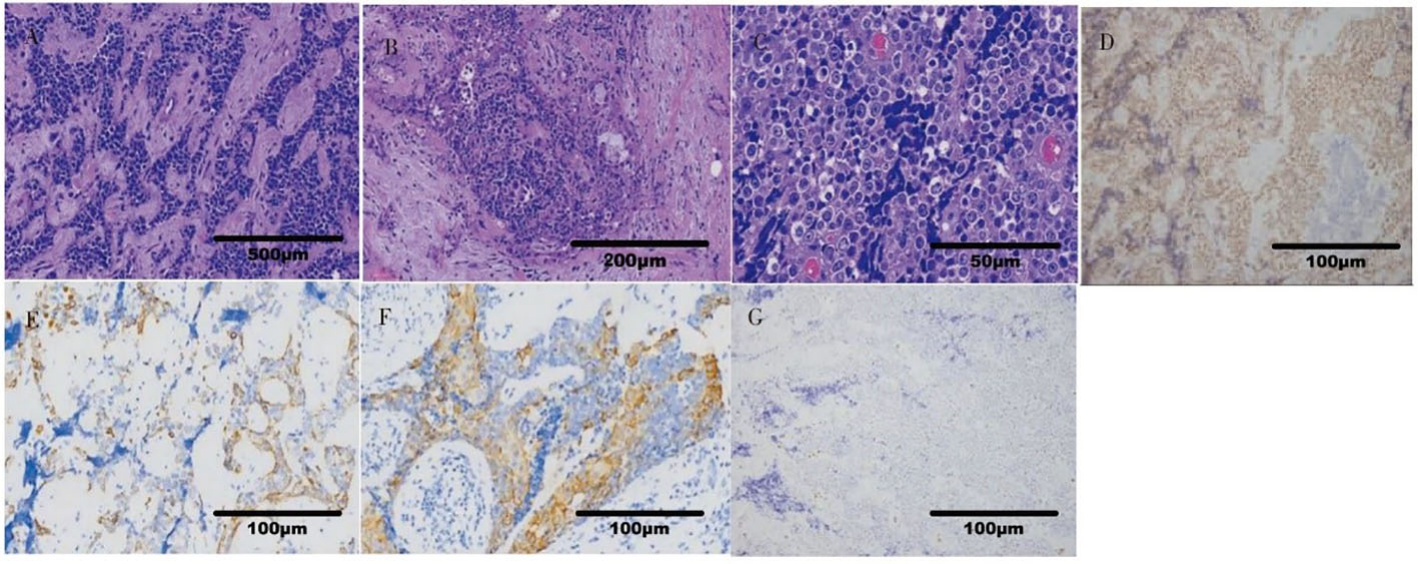
Histopathological findings of NUT carcinoma. **(A)** The tumor cells were distributed in nests (x40). **(B)** The structure was similar to that of lymphoepitheliomatoid carcinoma (x100). **(C)** The tumor cells had large nuclei, abnormal karyotypes, some nucleoli and occasional mitotic figures (x400). **(D)** NUT(+) (x200). **(E)** AE1/AE3 (partial+) (x200). **(F)** Squamous tumor cells EMA (partial+) (x200). **(G)** F1i-1 (individual+) (x200).

**Figure 2:**
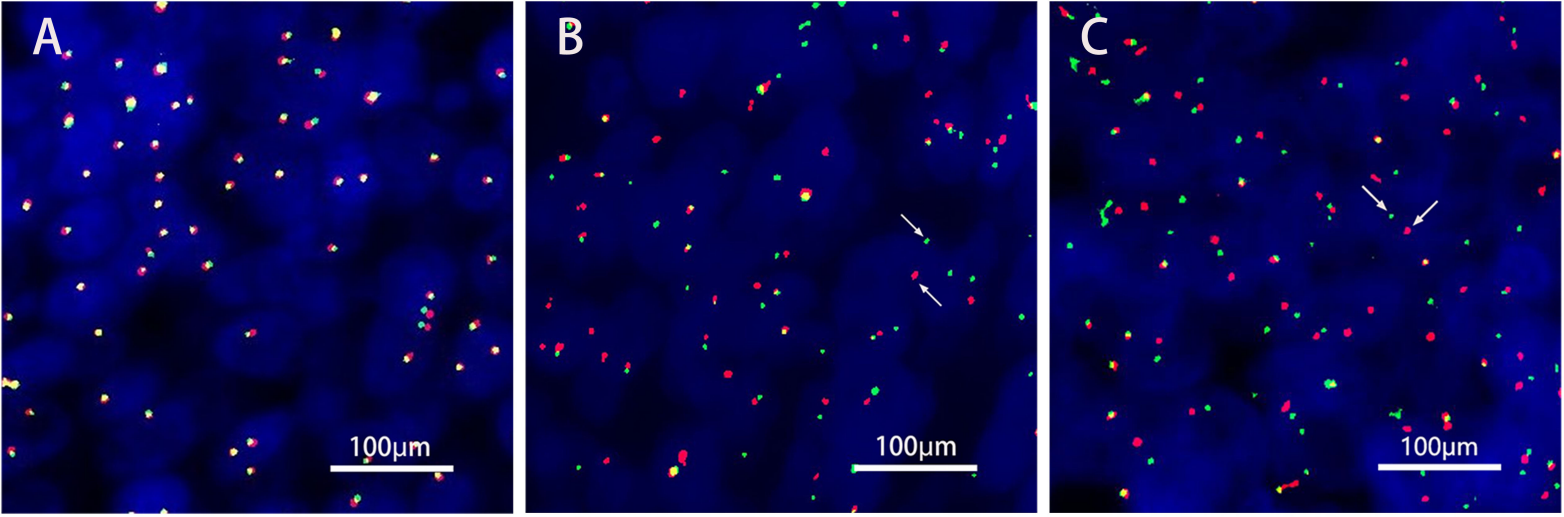
NUT gene rearrangement detection(FISH- Right parotid gland tissue). The green signal(G) was GSP NUT(Telomere); The red signal^®^ was GSP NUT(Centromere). **(A)** Negative control. **(B)** Positive control. **(C)**. FISH NUT (+) (red and green signal separation was seen in tumor cells, indicating that the gene was disrupted, Each signal mode is as follows: 1G1R1F 64.0%,IG1F 8.0%,1R1F 5.0%,1GIR 10.0%,2G1R 3.0%,IF 5.0%,2F 5.0%.).

A 36-year-old male patient came to the hospital in May 2020 with good health and no personal or family history of smoking. The patient complained that a tumor in the right parotid gland that had been found more than 3 years prior had gradually increased in early April 2020. Enhanced MRI of the parotid gland showed multiple lesions in the right parotid gland that might be considered adenocarcinoma (Warthin tumor). On May 19, 2020, right parotid gland mass resection and right parotid gland partial resection (superficial lobectomy) were performed under general anesthesia in our hospital. The pathological consultation results from the Ninth People’s Hospital Affiliated to Shanghai Jiao Tong University School of Medicine showed NUT carcinoma. Pathological results (8): Hematoxylin and eosin (HE) staining showed that the tumor cells were distributed in nests (**Figure 1A**). Lymphocytes in the stroma infiltrated in strips, similar to the structure of lymphoepitheliomatoid carcinoma (**Figure 1B**). The shape of tumor cells was irregular. Their cytoplasm was light eosinophilic red, and their nuclei were large. The karyotype was abnormal. Some nucleoli were seen, and mitotic figures were occasionally seen (**Figure 1C**). Immunohistochemistry results (8) (**Figures 1D–G**): NUT (+); AE1/AE3 (partial+); EMA (partial+); p63 (focal+); CK7, CK9, and MUC-1 slightly expressed; F1i-1 individual cells (+);CD56 and Syn (-). NUT gene rearrangement detection(FISH- Right parotid gland tissue): NUT gene break rearrangement detected, (**Figure 2**). Diagnosis: T3N0M0, phase III.

#### 2.1.2 Treatment

On June 12, 2020, PET-CT showed postoperative changes in the right parotid gland. Deep soft tissue thickening of the right parotid gland was accompanied by focal increased FDG uptake, with a diameter of approximately 1.5 cm. There was focal residual adenocarcinoma after right parotid gland surgery. Therefore, postoperative radiotherapy was performed in the Department of Radiotherapy, Renji Hospital Affiliated to Shanghai Jiao Tong University School of Medicine from June 29, 2020, and TP regimen chemotherapy combined with cetuximab targeted therapy (Abraxane 100 mg + DDP 40 mg + cetuximab 470 mg, intravenous infusion on the first day, once a week) was also administered. When the patient developed a grade I-II rash over his whole body (especially prominent on the skin of the nose) after 2 rounds of treatment, targeted therapy was discontinued. On July 17, 2020, and July 24, 2020, he received a third and fourth uneventful round of chemotherapy in our department. On July 30, 2020, MRI of the neck and clinical follow-up tests showed postoperative changes in the right parotid gland; specifically, the deep right-lobe lesion was significantly improved (less extensive and less diffusion restricted) compared to how it was on June 18, 2020. The efficacy assessment identified that the patient had achieved complete response (CR), and radiotherapy with 2 simultaneous chemotherapy sessions with the original regimen were administered (Aug 7, 2020, and August 14, 2020). The patient was subsequently clinically followed.

**Figure 3:**
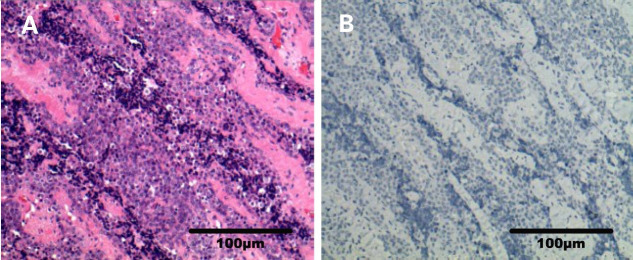
Photomicrograph of PD-L1 immunohistochemistry (right parotid gland). **(A)** Sample HE photomicrograph (x200). **(B)** Sample PD-L1 IHC photomicrograph (x200).

In February 2021, a physical examination revealed liver occupancy, and metastasis was considered. In March 2021, the patient went to Renji Hospital for radiofrequency ablation (RFA) of liver metastases. PET-CT on March 26, 2021, suggested that radiofrequency ablation of liver metastases did not deplete the lymph node metastases in the hilar region. A review of the liver MRI results on April 2, 2021, suggested small nodules at the junction of the hepatic S5 and S8 segments and periportal nodes, and the nodes showed evidence of tumor positivity. On April 15, 2021, the PD-L1 immunohistochemistry test results showed a tumor cell positive score (TPS) <1% and a combined positive score (CPS) <1 (**Figure 3**). On April 28, 2021, genetic analysis of the right parotid tissue showed the following: microsatellite stability (MSS), a tumor mutation load (TMB) of 4 Mut/Mb, and BRD4 exon 13 rearrangement. At the time, there were no appropriate clinical drugs or clinical trials based on the results. The TMB results suggested that the patient was unlikely to benefit from PD-1 or PD-L1 immune checkpoint inhibitor treatment. On May 6, 2021, MRI enhancement of the upper and middle abdomen was performed at Shanghai East Hospital: (1) Multiple occupancies in the liver, combined with the patient’s medical history and the change in the largest lesion in the right lobe after surgery, suggested metastasis. (2) There were multiple enlarged lymph nodes in the hilar area and retroperitoneum, and (3) chronic cholecystitis and ‘mud’-like stones in the gallbladder were present. From May 7, 2021, two courses of VAC+IE alternative chemotherapy were completed. The specific scheme is as follows: VAC (vindesine 3 mg iv gtt + epirubicin 135 mg iv gtt + cyclophosphamide 1.4 g iv gtt, q21d); IE (ifosfamide 2.3 g iv gtt + etoposide 0.1 g iv gtt d1-d3, q21d). On July 28, 2021, the images of enhanced MRI of the upper abdomen were reviewed at Shanghai East Hospital, and the efficacy assessment suggested no improvement. As such, the patient was recommended for enrollment in a clinical trial.

The results of an examination performed in September 2021 at Xiangya Hospital Central South University showed multiple nodules and masses in the liver and multiple positive lymph nodes in the hepatic hilum area and retroperitoneum. The short diameter of the larger lymph nodes was approximately 37 mm, and the size of the right anterior abdominal wall mass was approximately 38x27 mm. T5 vertebral body metastasis. The genetic testing results of the patient from April 28, 2021, showed BRD4 exon 13 rearrangement, and thus, the patient was given 1 tablet of NHWD-870HCI (a BET inhibitor (BETi)) orally each day from September 20, 2021.

On October 12, 2021, the patient came to our department with generalized jaundice of the skin and sclera. On October 15, 2021, our department conducted percutaneous transhepatic cholangiographic drainage (PTCD) to reduce jaundice. On October 19, 2021, the examination showed that the patient’s disease was progressing rapidly. Multiple intrahepatic masses were significantly enlarged and fused into a mass, with the maximum diameter of about 102.89mm. a right anterior abdominal wall mass (45.15 mm), multiple enlarged lymph nodes in the retroperitoneum of the abdominal cavity with localized invasion of the pancreas, and bone destruction of the thoracic 5 vertebrae by the surrounding soft tissue (19.45mm on the right, 10.66mm on the left). On October 21, 2021, we started zoledronic acid bone therapy. In November 19, 2021, the patient presented with sudden lower limb paralysis, and an urgent examination with CT suggested that the patient’s thoracic 4-6 vertebral body and accessory metastasis, with a paravertebral soft tissue mass, and part of the growth into the spinal canal, with spinal cord compression. Compared with the CT results from October 19, 2021, multiple intrahepatic masses were enlarged and fused into a mass (116.63mm), and the right anterior abdominal wall mass enlarged to 63.15 mm, the metastasis of thoracic vertebrae 4-6 (increase to 33.81mm on the right and 17.59mm on the left).

**Figure 4:**
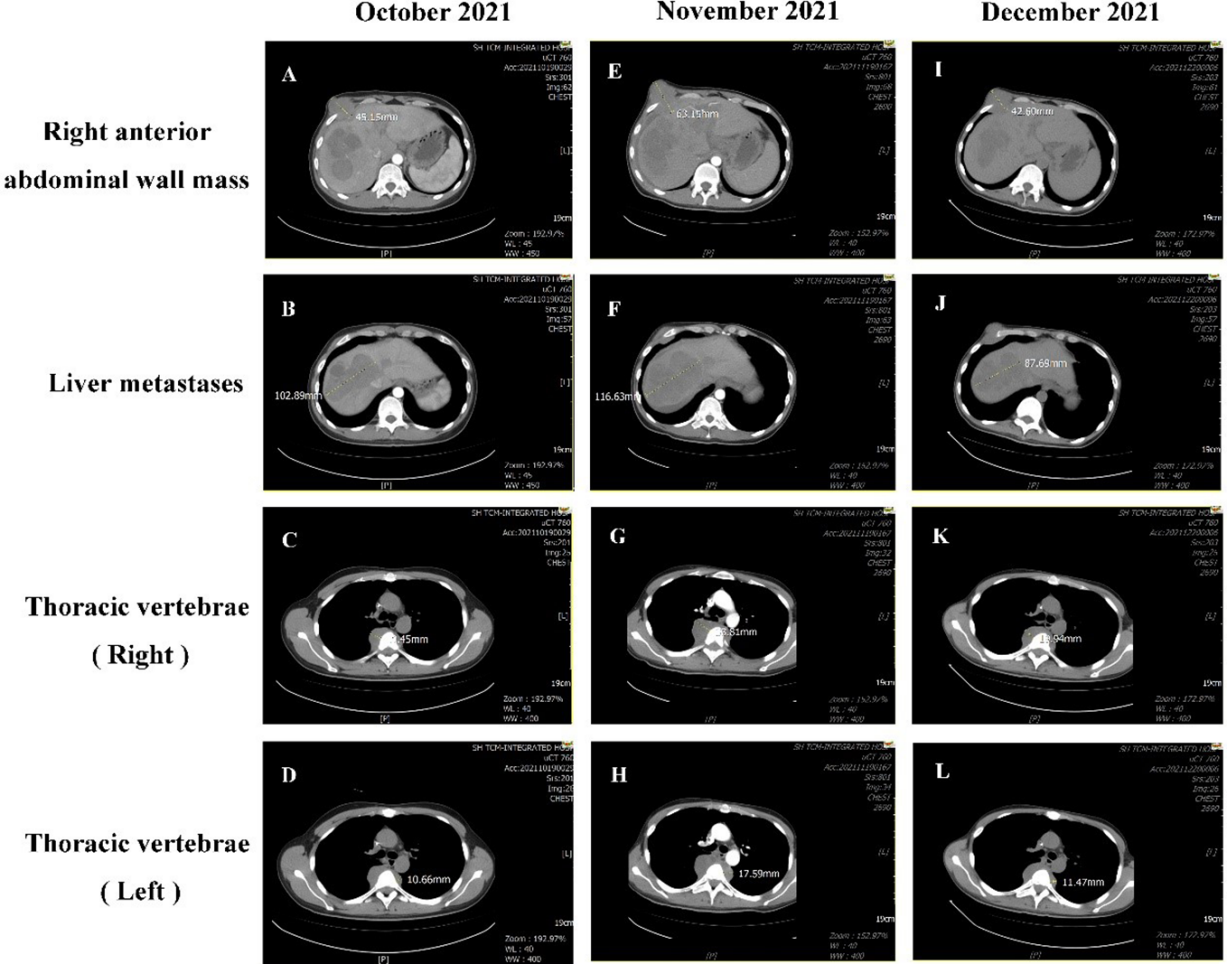
Comparison of CT results before and after treatment. On October 19, 2021, CT showed the following: **(A)** There was a right anterior abdominal wall mass approximately 45.15 mm in size. **(B)** Multiple intrahepatic masses were significantly enlarged and fused into a mass, with the maximum diameter of about 102.89mm.**(C)** The T5 soft tissue mass on the right side of the vertebral body approximately 19.45 mm in size. **(D)** The size of the T4-6 vertebrae metastasis on the left was approximately 10.66mm. On November 19, 2021, CT showed the following: **(E)** the right anterior abdominal wall mass had grown to 63.15 mm. **(F)** The size of the liver metastasis increased to 116.63 mm. **(G)** The T5 soft tissue mass on the right side of the vertebral body had increased to 33.81mm. **(H)** The mass on the left side of the T4-6 vertebrae was approximately 17.59mm. On December 21, 2021, CT showed the following: **(I)** The right anterior abdominal wall mass had decreased in size to 42.60 mm. **(J)** The sizes of the multiple masses in the liver were reduced to 87.69 mm. **(K)** The soft tissue mass surrounding the T5 vertebrae was 19.94 mm. **(L)** The mass on the left side of the T4-6 vertebrae was approximately 11.47 mm.

**Figure 5:**
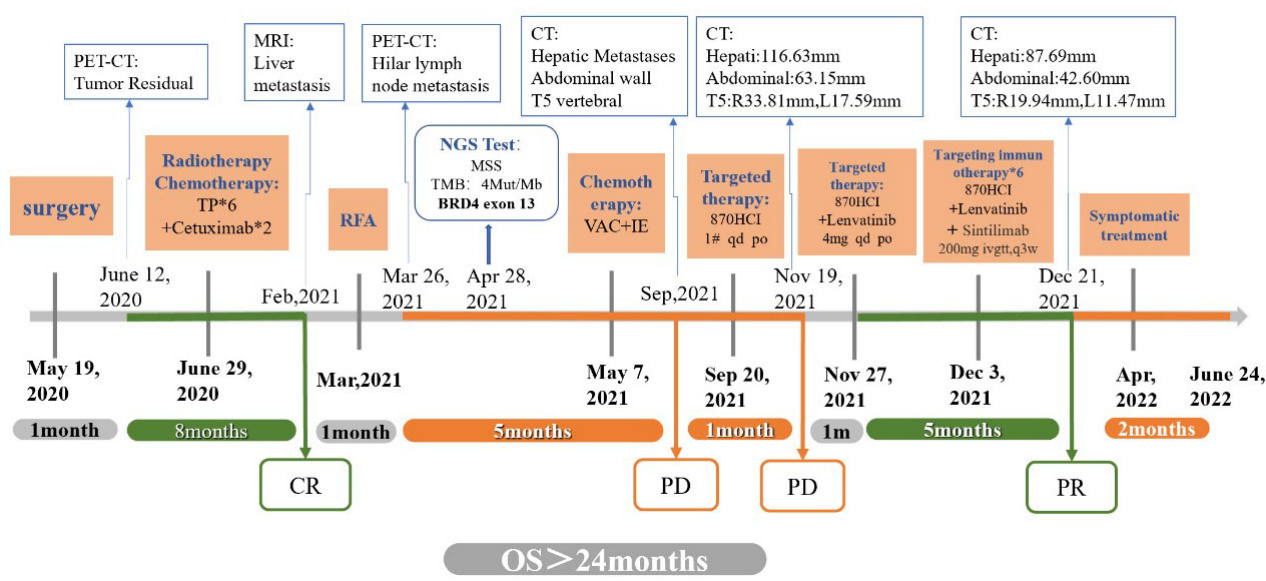
Timeline. The bold black text above the time axis indicates the time point of each examination; the bold blue text below the time axis indicates the time point of each treatment.

After communication and consent with the patient, targeted treatment with low-dose lenvatinib (4 mg qd po) was started on November 27, 2021. After contraindications for use were excluded, the first course of immunotherapy with sintilimab 200 mg iv gtt was performed on December 3, 2021. Contrast-enhanced CT was performed on December 21, 2021, to evaluate the efficacy. The enhanced CT showed that the right anterior abdominal wall mass was reduced to 42.60 mm, multiple intrahepatic masses were reduced to 87.69 mm, and the metastasis of thoracic vertebrae 4-6 (19.94 mm on the right and 11.47 mm on the left). The curative effect was evaluated as PR (**Figure 4**). On December 24, 2021, the patient was treated with a second course of sintilimab 200 mg iv gtt. The patient received a total of 6 courses of the NHWD-870 + lenvatinib + sintilimab combination regimen, and the disease was stable. In April 2022, the patient’s condition deteriorated, and he stopped receiving this regimen. On June 24, 2022, the patient died of multiple organ failure caused by extensive metastasis of the NUT carcinoma (**Figure 5**).

### 2.2 Discussion

#### 2.2.1 NUT carcinoma

NUT carcinoma is extremely rare and highly invasive. Currently, the etiology of the disease and its relationship with smoking or Epstein–Barr virus (EBV) infection are not clear. A review of the literature revealed a total of only 300 cases of NUT carcinoma of the head and neck (9), and only 15 of these cases have been parotid gland NUT carcinoma (7). Patients with salivary gland NUT carcinoma range in age from 12 to 55 years (median age 29 years) (10). NUT carcinoma progresses rapidly and features a high degree of malignant invasion, with a median survival time of 6 to 9 months. Eighty percent of patients die within 1 year after a definite diagnosis (11). Although the standard treatment for NUT carcinoma of the head and neck has not been established, multimodal treatment with systemic chemotherapy, surgery and radiotherapy has been applied clinically. Surgery is generally considered the best choice and is related to improved prognosis. Compared with radiotherapy or chemotherapy, complete surgical resection can significantly improve the survival rate, but a curative effect is not guaranteed. Most patients still need radiotherapy or chemotherapy after surgery (12). In particular, the application of cisplatin, paclitaxel and alkylating agents has achieved some benefits in clinical studies (13–15).

#### 2.2.2 Targeted therapy and immunotherapy for NUT carcinoma

Immunotherapy has been an important breakthrough in cancer treatments in recent years, and has provided novel strategies for tumor treatment that prolong the survival of patients. However, immunotherapy has a low efficacy rate, can take a long time to produce results, and may result in more serious and adverse reactions. To overcome these deficiencies of immunotherapy, immunotherapy can be combined with other treatments. Strategies combining immune checkpoint inhibitors with targeted therapy drugs have become some of the most popular immunotherapy strategies in recent years.

##### 2.2.2.1 Combination of BETis and immunotherapy

After the disease progressed in our patient, NHWD-870 (BETi) treatment was started, but poor efficacy was observed after 2 cycles. Studies have shown that there are two agents targeting the NUT-BRD4 fusion gene: histone deacetylase inhibitors (HDACis) and BETis. Although these two types of targeted agents show initial efficacy, all patients with head and neck NUT carcinoma treated with HDACis or BETis develop drug resistance and recurrence during treatment (16). NHWD-870 is a new type of BETi. Some researchers have found that NHWD-870 can improve the tumor immune microenvironment and enhance the efficacy of immunotherapy by blocking the tumor-macrophage interaction. Studies have shown that the combination of immune checkpoint inhibitors and BETis can prolong the survival time of animals with melanoma by up to one year (17). BETis have a better effect when used in combination with anti-PD-1/PD-L1 antibodies or other targeted drugs. Based on this, after 2 cycles of treatment with NHWD-870 alone, we treated the patient with 2 cycles of a PD-1 inhibitor combined with andomized, and partial response (PR) was achieved.

##### 2.2.2.2 Small-molecule tyrosine kinase inhibitors combined with immunotherapy in the treatment of NUT carcinoma

Considering the poor efficacy of the BETi alone, the patient received NHWD-870 + andomized + a PD-1 inhibitor for 2 cycles. Immunotherapy combined with targeted therapy has been the focus of antitumor research in recent years. The KEYNOTE-524 study showed that the objective remission rate (ORR) of patients receiving Keytruda combined with andomized (the “cola combination”) as first-line treatment for advanced hepatocellular carcinoma (HCC) was 46% (18). The ORR of patients receiving the PD-L1 immune checkpoint inhibitor atezolizumab combined with the antiangiogenic drug bevacizumab (“A+T”) as first-line treatment for unresectable HCC was 33.2% (19). The ORR of patients receiving sintilimab combined with bevacizumab as first-line treatment for unresectable or metastatic HCC was 23.4% (20). However, there is still a lack of understanding about the basic pathophysiological mechanisms of combined therapy. Some studies have shown that antiangiogenic drugs can have a synergistic effect with immune checkpoint inhibitors because they can improve the tumor immune microenvironment (21). However, the specific mechanism underlying the improvement in the tumor immune microenvironment is not clear, and little is known about the molecular mechanisms of small-molecule multitarget TKIs, such as andomized. In May 2021, a multidisciplinary team from the Department of General Surgery, Gastroenterology and Infectious Diseases of Huashan Hospital affiliated with Fudan University explained the mechanism by which andomized regulates the immune microenvironment. During the study, the team found that the expression of PD-L1 decreased in two patients with liver cancer that recurred in a short time after surgery after taking ranvastinib for about two months. It has been confirmed in animal and *in vitro* experiments that andomized can indeed downregulate the expression of PD-L1 in HCC cells (22). Subsequent animal experiments showed that andomized downregulated PD-L1 expression mainly by inhibiting the FGFR4 signaling pathway. In addition to targeting FGFR4, andomized can inhibit the differentiation of regulatory T cells (Tregs), thus blocking the inhibitory effect of Tregs on anti-PD-1 monoclonal antibody therapy and resulting in significantly improved therapeutic effects. These results not only provide a better understanding of the interaction between targeted drugs and immunosuppressants but also lay a solid foundation for the subsequent clinical application of combination regimens with targeted drugs and immunotherapy.

Although the response of patients to andomized combined with anti-PD-1 monoclonal antibody treatment is excellent, the regimen still has a limited scope of application and is more suitable for patients with high FGFR4 expression and Treg infiltration. However, more rigorous large phase III randomized controlled trials are needed. The LEAP-005 study published by ASCO investigated the use of andomized combined with an anti-PD-1 monoclonal antibody as treatment for multiple tumors. The results showed that the combination achieved good results for multiple tumors. When used as later-line therapy for non-small cell lung cancer (NSCLC), the ORR was 33%; when used as second-line treatment for biliary tract tumors, the disease control rate (DCR) reached 68%; and when administered to patients with PD-1 inhibitor-resistant melanoma, the OS was more than 1 year.

In summary, reviewing the treatment of this case, from the published results, we can see that the treatment of PD-1 monoclonal antibody combined with andomized shows a positive treatment effect in cases of multiple tumors. Given that preclinical studies have shown that BETis and immune checkpoint regulators have a synergistic effect because they regulate the expression of the immune checkpoint ligand PD-L1, we administered NHWD-870 + andomized and a PD-1 inhibitor. The number of clinical trials studying immunotherapy and targeted immunotherapy combinations is increasing. For patients with BRAFv600 mutation-positive, unresectable or metastatic melanoma, the FDA approved the first immunotherapy + dual-targeting triple drug regimen on July 30, 2020: the PD-L1 inhibitor atezolizumab + the MEK1/2 inhibitor cobimetinib + the BRAF inhibitor vemurafenib. We suggest that targeted therapy combined with immunotherapy which has long-term clinical benefits and targeted therapy which has high clinical response rate(immunotherapy + dual-targeting three-drug regimens) is an ideal choice for the treatment of patients with rare and/or refractory tumors and will not compromise patient safety. Reflecting on the treatment process of this case, we regret that we did not get the pathological tissue of liver metastasis for gene detection again. In addition, at present, many targeted preparations such as HDACi, BETi and CDK9 are still in clinical trials. The lack of access to these latest drugs for treatment also limits the possibility of more benefits for this case.

## Data availability statement

The original contributions presented in the study are included in the article/supplementary material. Further inquiries can be directed to the corresponding author.

## Ethics statement

The studies involving human participants were reviewed and approved by IRB of Shanghai TCM-Intergrated Hospital, Shanghai University of TCM. The patients/participants provided their written informed consent to participate in this study.

## Author contributions

SF and ZW contributed equally as first authors. SF, CL and YL contributed to the conception or design of the work, or the acquisition, analysis, or interpretation of data for the work. ZW, ZZ and KZ involved with drafting the work or revising it critically for important intellectual content. SF and ZW made final approval of the version to be published. YZ was accountable for all aspects of the work in ensuring that questions related to the accuracy or integrity of any part of the work are appropriately investigated and resolved. All authors contributed to the article and approved the submitted version.
